# *Pinguicula rosmarieae* Casper, Bussmann & T.Henning (Lentibulariaceae), a new butterwort from the Amotape-Huancabamba Zone (northern Peru)

**DOI:** 10.3897/phytokeys.140.49529

**Published:** 2020-03-04

**Authors:** S. Jost Casper, Rainer W. Bussmann, Tilo Henning

**Affiliations:** 1 Waldpark Seniorenpflegeheim, Prellerstraße 16, D-01309 Dresden, Germany Waldpark Seniorenpflegeheim Dresden Germany; 2 Department of Ethnobotany. Institute of Botanu, Ilia State University, 1 Botanical Street, 0105 Tbilisi, Georgia Ilia State University Tbilisi Georgia; 3 Freie Universität Berlin, Botanischer Garten Botanisches Museum, Königin-Luise-Str. 6-8, D-14195 Berlin, Germany Freie Universität Berlin Berlin Germany

**Keywords:** Lentibulariaceae, *
Pinguicula
*, *Pinguicula rosmarieae*, Peru, Amotape-Huancabamba Zone, Cordillera Central, endemic, taxonomy, new species, distribution

## Abstract

The insectivorous genus *Pinguicula* occurs along the whole Andean mountain chain from Colombia-Venezuela in the north to Tierra del Fuego in the south with a short interruption in the Peruvian-Chilean desert range. This paper describes a new and striking species of *Pinguicula* that occurs in the south-eastern part of the Amotape-Huancabamba Zone in north Peru. It grows either as a lithophyte on moist rocks or as an epiphyte on *Polylepis multijuga* Pilg. in the wet highlands of the Cordillera Central. *Pinguicula rosmarieae* Casper, Bussmann & T.Henning, **sp. nov.** is clearly distinguished by a basal rosette of ovate-obovate leaves spread out flat on the ground and especially by a two-partite corolla with a straight uniform tube-spur complex, two features unknown from other Andean *Pinguicula* species. The morphological similarity to *P. calyptrata* Kunth is discussed and the habitat and distribution of *P. rosmarieae* are characterised.

## Abstract (es)

El género insectívoro *Pinguicula* se encuentra a lo largo Andes desde Colombia y Venezuela en el norte hasta Tierra Fuego en el sur, con una breve interrupción en el los desiertos peruano-chilenos. Este artículo describe una nueva y distintiva especie de *Pinguicula* que se encuentra en la parte sur de la zona Amotape-Huancabamba en el norte del Perú. Puede crecer tanto como litófita sobre rocas húmedas o como epífita sobre *Polylepis multijuga* Pilg. en las tierras altas y húmedas de la Cordillera Central. *Pinguicula rosmarieae* Casper, Bussmann & T.Henning, **sp. nov.** se distingue claramente por tener una roseta basal de hojas ovadas-obovadas, postradas sobre el suelo y, especialmente, por la corola bipartida con un espolón uniforme recto, una combinación de características desconocidas de otras especies andinas de *Pinguicula*. Se discute la similitud morfológica con *P. calyptrata* Kunth y se caracterizan el hábitat y la distribución de *P. rosmarieae*.

## Introduction

In South America, the insectivorous genus *Pinguicula* (Lentibulariaceae) is represented by seven at first sight ± closely related, although not well-known taxa: *P. antarctica* Vahl, *P. calyptrata* Kunth, *P. elongata* Benj., *P. involuta* Ruiz & Pav., *P. jarmilae* Halda & Malina, *P. nahuelbutensis* Gluch and the here newly described *P. rosmarieae* Casper, Bussmann & T.Henning. Forming a nearly continuous chain of ± vicarious species, the taxa occur over a distance of 8,500 km in the Andes from the north (Colombia, Santa Marta) via Venezuela, Ecuador, Peru, Bolivia and Chile to the extreme south (Chile, Argentina: Tierra del Fuego), with a gap between ~30°S and ~36°S in the Peruvian-Chilean arid region. This “Atacama”-gap has been bypassed (perhaps in former cooler and wetter periods). In northern Peru (~ 05°30'–06°30'S), the ‘páramo-butterwort’ *P. calyptrata* is more or less replaced by the litho-/epiphyte *P. rosmarieae* and, further to the south, the ‘Jalca-Puna’-butterworts *P. involuta* and *P. jarmilae* close the gap.

## Collection history

In 2017, searching for material of *P. involuta* during his study of the South American *Pinguicula* taxa, the first author came across the Catalogue of the Flowering Plants and Gymnosperms of Peru (Peru Checklist) (https://www.tropicos.org/Project/PEC). At first sight, he believed that the specimen MO 6607881 (Paniagua Zambrana, Bussmann & Vega Ocaña 8586), designated as *Pinguicula*, could represent a new species, but more information, especially photos and, ideally, additional material was needed to confirm this initial assumption. The plants were gathered in November 2012 in the eastern Andes of north Peru in the area surrounding the Laguna Huayabamba (“Huayllabamba”) and described as growing on a vertical rock wall in the spray of a small waterfall. The collectors located the sampled population in the Department La Libertad, but, instead, they were already in the adjacent Department San Martín.

Further investigations drew the attention to another, earlier collection (May 2001: T. Henning and C. Schneider 275) deposited in the Berlin herbarium (B) at the BGBM. They collected a violet-white flowering *Pinguicula* growing in large stands on moist rock surfaces in the cliffs forming the southern limit of the Laguna de los Cóndores (~06°51'S, ~77°42'W), ca. 20 km east of Leymebamba (B 100136109, B 100136110; duplicate specimens in HUT). The original label indicates “Departamento Amazonas, Province Chachapoyas”. However, the Laguna and the adjacent area are part of the Department San Martín and situated some 4–5 km east of the border to the Department Amazonas. These specimens enabled the first author to conduct morphological comparisons with the material at MO for a thorough investigation. The collections showed the same taxon from a different population some 15 km further north in the same mountain range. The morphological differences to the other Peruvian *Pinguicula* species proved to be stable, at least in these two populations sampled independently and, together with the reported peculiar habitat preferences, enough to justify the description of a new species.

Finally, just very recently (2019), a third population has been reported and documented photographically by Lázaro Santa Cruz Cervera (USM) from the province of Bongará in the Department Amazonas, some 100 km further north.

## Taxonomic background

During the process of describing the new species, the most important Peruvian herbaria in Lima (USM) and Trujillo (HUT) have been contacted in order to locate the duplicates and gather information about potential additional collections and records. After a review of all herbarium material that we could get access to, it became apparent that most of the *Pinguicula*-collections from north Peru have been misidentified as *Pinguicula involuta*. Instead, all collections made north of the Department Huánuco in central Peru belong to *P. calyptrata*, a species traditionally referred to as a Colombian-Ecuadorean taxon. This common misconception is due to the inadequate original description and the therein indicated distribution of these two Andean butterworts. Whereas *P. calyptrata* was collected by Humboldt and Bonpland (Humboldt et al. 1817) in Saraguro in the southern Ecuadorean province Loja, *P. involuta* was described by Ruiz and Pavon (1798) from Huánuco in central Peru and especially the latter is insufficiently characterised in the protologue (Casper and Hellwig 2019).

However, the distributional patterns revealed from the two previously described and the undescribed species draw a much clearer picture corresponding to the phytogeographic characteristics of the so-called Amotape-Huancabamba Zone (hereinafter: AHZ) (Weigend 2002). Instead of the (simplified) subdivision into a northern (*P. calyptrata*) and southern (*P. involuta*) taxon whose limits roughly correspond to the border between Peru and Ecuador, *Pinguicula* is present in the AHZ with two taxa, one occurring in the west stretching northwards (*P. calyptrata*) and one endemic to the eastern slope of the Peruvian Andes (*P. rosmarieae*). South of the AHZ, they are replaced by the widespread *P. involuta* whose distribution extends into Bolivia. The new taxon, *Pinguicula rosmarieae* Casper, Bussmann & T.Henning spec. nov. is here described as new to science. The morphology of the new species and its affinities to related taxa are illustrated and discussed. The coarse biogeographical patterns observed for the Peruvian species are outlined and explained in the context of the characteristics of the AHZ. A distribution map, based on collection data from revised herbarium material and a key to the Peruvian *Pinguicula*-species, is provided to enable a reliable determination of existing and future collections which is a crucial component of floristic studies as basis for urgent conservation efforts.

The present study is the first of a series of contributions at different stages of completion (Casper; Casper et al. in prep.), each dealing with a certain taxonomic or nomenclatural problem that became apparent in anticipation of a comprehensive synopsis of the South-American *Pinguiculas* that is in preparation and will be published soon. Therein, all questions regarding the historical biogeography as well as nomenclatural and taxonomic issues will be discussed extensively.

## Materials and methods

The first author examined herbarium specimens of *Pinguicula* L. from South America in preparation for a revision of the Andean *Pinguiculas* at HUT, USM, MO, B and M (Thiers 2019) and from specimen scans using online databases (www.tropicos.org). This study combines the results of the herbarium studies, the experiences of the collectors (R.W. Bussmann and T. Henning) in the natural habitat and observations and reports kindly received from the Peruvian colleagues.

## Results

### Key to the Peruvian species of *Pinguicula*

**Table:** 

1	Foliage not star-like, leaf blades oblong-obovate-ovate, with margins slightly (mostly ~2 mm) curled up; corolla bi-partite, i.e. divided in the lip and the straight, more or less uniform tube-spur complex (the funnel-shaped tube merges into the conical blunt spur, with little to no angle); living on water-rinsed sandstone rocks or as epiphyte on *Polylepis* twigs; cloud forest (eastern North-Peru)	***P. rosmarieae***
–	Foliage star-like (“stellate”), leaf blades ovate, with margins distinctly curled up (appearing boat-shaped); corolla tri-partite, i.e. divided distinctly in lip, tube and spur, i.e. the tube distinctly angled with the spur	**2**
2	Corolla with nearly equal-sized notched lobes (subisolobate, i.e. lobes of the upper-lip only slightly smaller than those of the lower lip); ~2 mm behind the lower lip middle lobe base, a prominent clapper-like palate covered by yellow hairs inserted; the tube typical funnel-shaped; the spur short, conical, blunt; páramo–jalca (North-Peru)	***P. calyptrata***
–	Corolla with distinctly unequal-sized notched lobes, (i.e. lobes of the upper-lip lobes distinctly smaller than those of the lower-lip); the lower-lip middle lobe dominating the lip, often distinctly bent down; no distinct palate inserted; the tube cylindrical, nearly as long as wide; the spur slender, sickle-shaped, pointed; puna (Central- and South-Peru)	***P. involuta***

### Taxonomic treatment

#### Pinguicula rosmarieae

Taxon classificationPlantaeLamialesLentibulariaceae

Casper, Bussmann & T.Henning sp. nov.

D41B4E95-A94B-5E22-A7A5-7B38BAAF4A9B

urn:lsid:ipni.org:names:77206951-1

Figs 1 A–H , 3A

##### Type.

**Figure 1.**
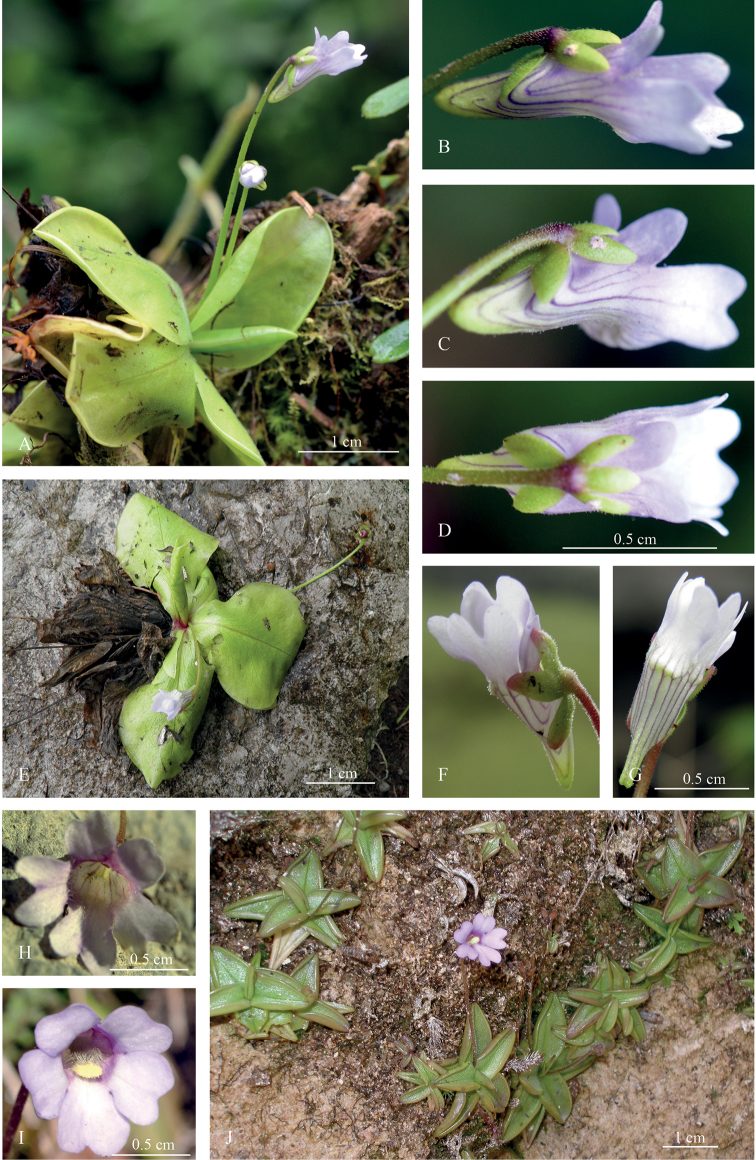
*Pinguicula rosmarieae* (**A–H**) *and P. calyptrata* (**I, J**). **A***P. rosmarieae*, 2-scaped; upper flower opened, in profile view, lower one in bud; epiphytic on *Polylepis multijuga* Pilg., in *Polylepis multijuga*-*Iochroma stenanthum* S.Leiva, Quip. & N.W.Sawyer – dominated cloud forest. Peru, Department San Martín, close to ‘El Jardín’(Inca-hut and surrounding area east of the Laguna Huayabamba), 3,090 m a.s.l., 06°56'044"S, 077°41'54"W**B** ditto, flower, profile view **C** ditto, flower, semi-ventral view **D** ditto, flower, dorsal view **E***P. rosmarieae* rosette from the northernmost known habitat, “Hatumpampa” Department Amazonas, Province Bongará (no voucher specimen) **F** ditto, flower, semi-ventral view **G** ditto, flower ventral view **H** corolla in frontal view, lower-lip lobes to ¼ of its length notched, throat without distinct palate. Peru, Department San Martín, Laguna de los Cóndores (Henning & Schneider 275) **I***P. calyptrata*, corolla in frontal view, lobes with lateral margins slightly covering each other, lower-lip lobes to 1/6 of its length notched, throat with clapper-like yellow palate. Peru, Department San Martín, Sphagnum-bog, 3,000 m a.s.l. above the Laguna de los Cóndores (Bussmann, A. Glenn, G. Chait & C. Vega Ocaña 16447) **J** flowering stand of *P. calyptrata* near Pulan, Cajamarca. (Credits: photographs **A–D, I** R. W. Bussmann **E–G, J** L. Santa Cruz Cervera **H** T. Henning).

**Figure 2.**
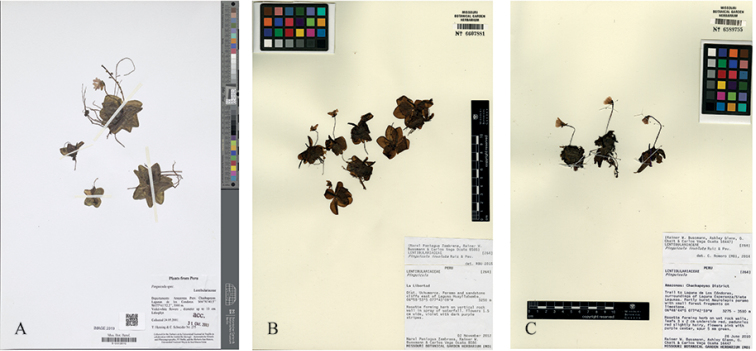
Specimen scans of *Pinguicula rosmarieae* and *P. calyptrata*. **A** Isotype of *P. rosmarieae* – Scan (Henning & Schneider 275, B100136110), with permission **B** paratype, from vertical sandstone cliff east of the Laguna Huayllabamba, 3,250 m a.s.l., the three flowers (left) showing corollas with their significant straight uniform tube-spur-complex. The rosette is composed of flat outspread leaves. – Scan of MO 6607881, with permission **C***P. calyptrata*, the leaves have curled up margins and the corollas are typical tri-partite in corolla, tube and spur. The corolla lobes are nearly equal-sized, the tube funnel-shaped and the spur short and angled with the tube. – Scan MO 6589755; with permission.

**Figure 3.**
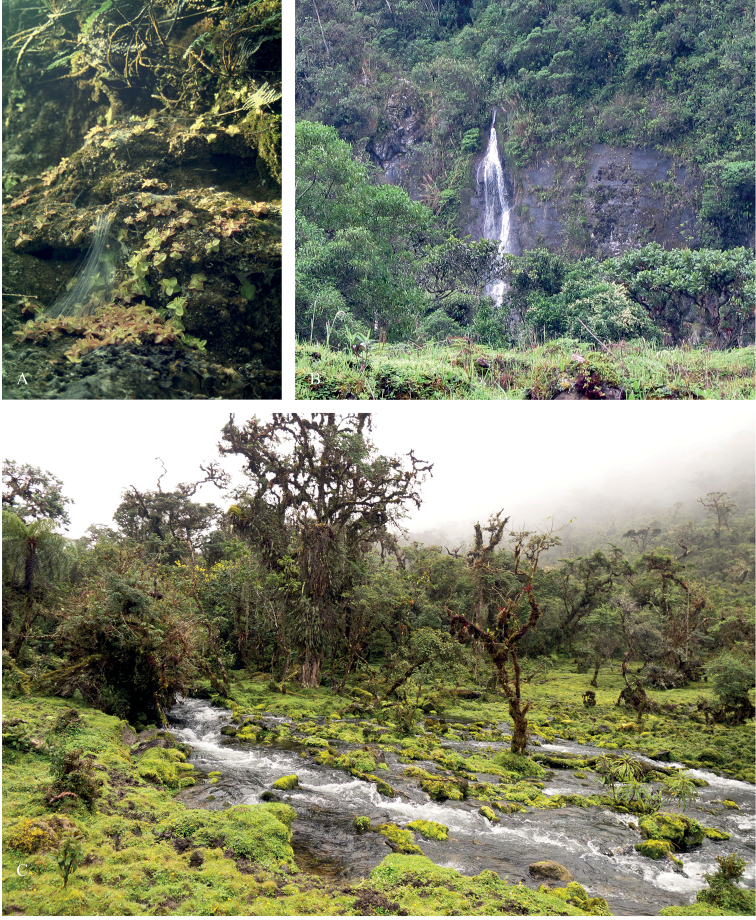
Habitats of *Pinguicula rosmarieae* in the Department San Martín. **A** Large stands at the type locality above the Laguna de los Cóndores **B** sandstone rock walls with small waterfall near ‘El Jardín’ **C** ‘El Jardín’ *Polylepis multijuga* stands with *P. rosmarieae* growing as an epiphyte. (Credits: **A** T. Henning, **B, C** R. W. Bussmann).

Peru: Dept. San Martín, Prov. Huallaga [“Dept. Amazonas, Prov. Chachapoyas” (sic!)], Lagunas de los Cóndores (06°50'40.5"S, 77°41'52.2"W), 3,000 m a.s.l. Violet-white flowers, diameter up to 100 mm. Lithophyte, 24 May 2001, T. Henning & C. Schneider 275. (***Holotype***: HUT 41126!: det. Pinguicula spec. – Figs 1H, 3A; ***Isotypes***: B: B 100136110 (Fig. 2A)! B 100136109!)

##### Diagnosis.

Herba perennis rosulata, rosula ca. 100 mm in diametro; lamina foliorum circuito suborbiculata vel obovata, margine vix (~3 mm) involuta, ca. 30–40 mm longa ac lata, solum plusminusve adpressa; scapus 1(–4), erectus, 20–40 mm altus, teres; flores 1, parvi, ~8–10 mm longi (tubo-calcari incluso), bilabiati; calyx lobis oblongis, lobis superis usque ad basin fere divisis, lobis inferis usque ad dimidium divisis, ad angulum ~45° divaricatis; corolla lobis 5, oblongis, ~5–7 mm longis, subisolobatis, apice valde emarginatis; corollae tubum infundibuliformi-cylindraceum cum calcari conico ± uniformem rectum coniunctionem formans, 5–6 mm longum apice obtusum.

Habitatio in locis apertis et humidis montium Andinensium regionis Peruviae septentrionalis usque ad 3.100 m supra mare, praesertim ad rupes et saxa. Habitu Pinguiculae Andinensium simili, praecipue differt tubo-calcari-coniunctio recto uniformi.

##### Description.

Perennial rosette leaved herb with 1 (–4) flowered scapes. *Rhizome* ~ 10 mm long, with numerous adventitious fibrous roots. *Leaves* (4–) 6–10, flat on the ground, ± succulent (dried translucent-membranous), adult (20–) 30–40 (–50) mm long, nearly as long as wide, the blades ovate-obovate-oblong in outline, rounded at the tip attenuated to the base into a short petiole, the margins weakly (up to 3 mm) curled up, yellowish-green, upper surface of lamina covered with sessile glandular hairs. *Hibernacula* (winter buds, dormant buds) absent. *Scapes* 1–2 (–4), erect, (20–) 30 (–40) mm tall, terete, filiform (0.5–1 mm thick), one–flowered, green to reddish-brown, scattered with glandular hairs, often becoming glabrous or nearly so. *Flowers* small, ~8–10 (–11) mm long (including tube-spur-complex). *Calyx* two-lipped, green to pale brown to purple, upper surface of sepals scattered with stalked glandular hairs; upper lip divided deeply into three nearly equal-sized oblong lobes, at apex pointed; lower lip up to ½ divided into two oblong lobes, at apex pointed. *Corolla* two-lipped, bluish-magenta to white-violet; upper lip two-lobed, lobes obovate, ~5–6 mm long and ~2–3.5 mm wide, shallowly notched at the apex; lower lip larger and longer than the upper lip, with three oblong to obovate-oblong lobes (the median lobe somewhat larger than the two lateral ones), 4.5–5 mm wide, each distinctly (~1/5 to 1/3 of its length) notched. *Tube* (*tube-spur-complex*) at the throat funnel-shaped, on both sides broader than the *spur*, on the back side higher than the spur, proximally cylindrical (nearly as long as wide), on the ventral side merging without any sharp angle into the cylindrical to cone-like stubby, at apex rounded, yellow-greenish spur; tube and spur forming a more or less uniform funnel- to cone-like straight, from the ventral side appearing as a box-like ‘*tube-spur-complex*’, ~6 mm long; the tube-spur-complex externally dark blue to purple lengthwise-striped by parallel veins. *Palate* bipartite, weakly developed (not clapper-like), inserted immediately behind (~1–2 mm) the corollas´ lower-lip middle lobe, yellow, set with short-stalked glandular hairs, proximally elongated into a short ventral hair strip; each of the two lateral corolla lobes with a small yellow hair bubble at their base, stretching proximally along on each side of the inner tube wall. *Indumentum* (apart from that visible in the photographs), *stamens, pollen grains*, *ovary, stigma, capsule, seeds* not seen. *Chromosome number* unknown.

**Figure 4.**
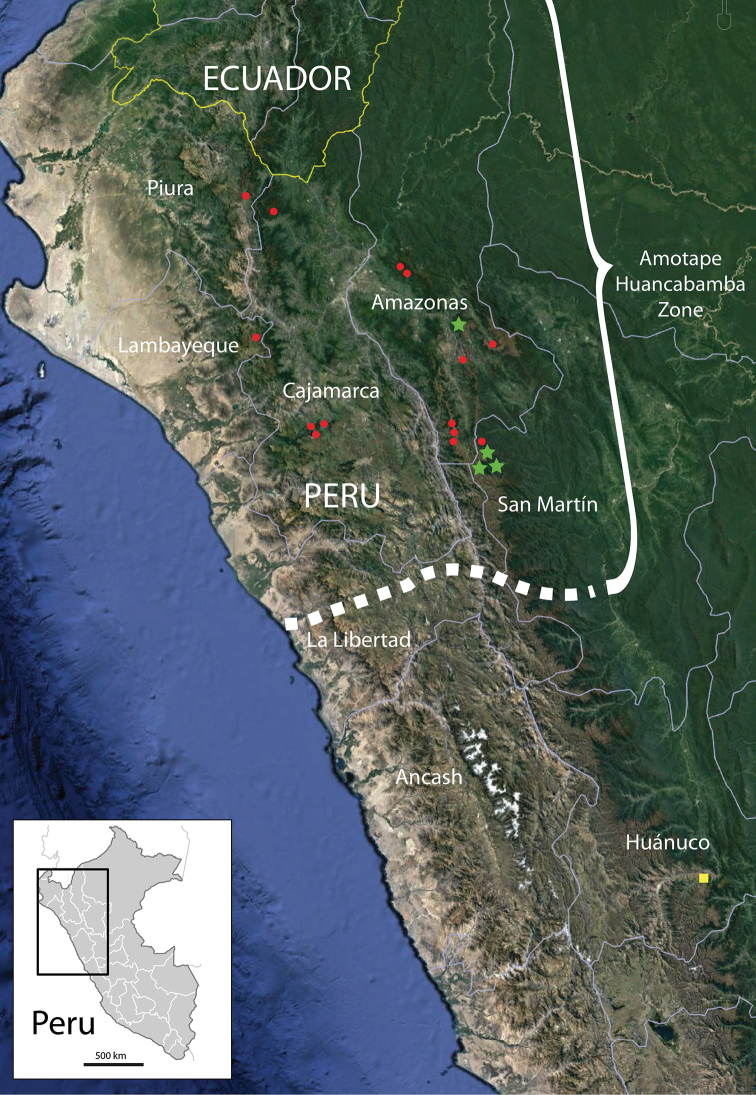
Distribution of *Pinguicula rosmarieae* and related taxa based on geo-referenced collections. Green stars: *P. rosmarieae*, red dots: *P. calyptrata*, yellow square: *P. involuta*, dotted line: northern and southern limits of the Amotape-Huancabamba-Zone.

*Pinguicula rosmarieae* is distinguished by its notable uniform funnel-cone-shaped straight tube-spur-complex, a feature unknown in any other Andean *Pinguicula* taxon. It is an endemic species, restricted to the eastern slopes of the Cordillera Central within the Amotape-Huancabamba-Zone of northern Peru (Departments Amazonas and San Martín; Fig. 4). It grows in rocky habitats, either under moving water or in the spray of small waterfalls and occasionally epiphytic on moss-covered twigs of *Polylepis multijuga*, at altitudes of about 2500 m–3100 m a.s.l. (Fig. 3).

##### Etymology.

The new species is named after Dipl.-Biol. Rosmarie Casper, beloved wife and steady companion of the scientific efforts of S. J. Casper and mother of their children.

## Discussion

### Affinities

Based on current floristic literature, *Pinguicula involuta* was considered as the only butterwort native in northern Peru. However, a revision of the available herbarium material in this study has revealed that this simple assumption, according to the historical taxonomic treatments, *P. involuta* is native to Peru whilst *P. calyptrata* is native to Ecuador, is largely incorrect. Furthermore, the “northern” *Pinguicula* of Humboldt and Bonpland (i.e. *P. calyptrata*), that was first discovered in the Saraguro range in southern Ecuador, has repeatedly been collected in northern Peru. We have seen specimens of *P. calyptrata* from the Peruvian Departments Piura, Amazonas, San Martín, Lambayeque and Cajamarca (Fig. 4, cf. the list of specimens below). In turn, *P. involuta*, so far, has never been collected in these Departments, although misidentified collections and erroneous floristic literature suggest that. Based on our observations and fieldwork, the northernmost locality of *P. involuta* is in the Department Huánuco in central Peru, some 350 km further south of the southernmost *P. calyptrata* localities (Province Pachitea, Panao; road from Chaglia [Chaglla] to Rumichaca [Tambo de Vaca], km 81, 09°51'S, 75°53'W), leg. M. Weigend, K. Weigend, T. Henning, & Ch. Schneider 5426, Fig. 4). At present, the exact distribution limits of *P. involuta* and *P. calyptrata* remain uncertain.

Morphological data indicate that the new taxon is distinct from *P. involuta* and *P. calyptrata*. A thorough study of South American *Pinguicula* is in preparation and will elucidate the morphological affinities amongst all relevant taxa.

*Foliage*: The leaf blades of *P. rosmarieae* are ovate-obovate-oblong, dried membranous-translucent (Fig. 2A) and spread out, i.e. they are not boat-like and they lack heavily curled-up margins (Fig. 1A, E): The whole rosette (up to 100 mm across) is not star-shaped compared to *P. calyptrata* (Figs 1J, 2C) and *P. involuta* (field name of the latter in the original collection was *P. stellata*, see Casper and Hellwig 2019). The foliage shape of *P. rosmarieae* is similar to the geographically-distant *P. albida* Wright that occurs in Cuba and may represent an individual adaptation to the rocky, exposed habitats. Both habitats share nutrient-poor and often acidic white-sand-soils (for Peru: M. Weigend, pers. comm.). However, this observation is contradicted by the fact that the rosettes of *P. calyptrata* and *P. involuta* retain their star-like appearance on relatively open stands. Shape and posture of the foliage are, therefore, taxonomically meaningful for species delimitation.

*Corolla*: The violet-white to pale-bluish corolla is similar to that of most Andean *Pinguicula*: its lips are nearly equal-lobed (subisolobate). The two lobes of the upper-lip are (mostly only shallowly) notched at the distal margins (Fig. 1B–D, F–H), the three lobes of the lower-lip are longer and at its distal margins deeply notched (up to ¼ –1/3 of its length; Fig. 1G–H). The most striking feature that separates *P. rosmarieae* from other Andean *Pinguicula* taxa is the more or less uniform straight tube-spur-complex that appears as almost entire. The typical tri-partite divided *Pinguicula* corolla (i.e. into corolla lobes, tube and spur) appears nearly bi-partite here and is divided only into the corolla lobes and the tube-spur complex. The funnel-shaped, cylindrical tube merges ventrally into the short and comparatively wide cone-shaped spur, lacking a sharp angle (Fig. 1A, B). The spur is stubby, rounded at the apex and light yellow-green. The back of the tube only weakly protrudes from the spur: from this point, the stubby spur slightly attenuates proximally and ends in an obtuse apex. Looking at the corolla from a lateral (Fig. 1B) or semi-ventral (Fig. 1C) perspective, the tube and spur are not markedly separated. The tube-spur-complex is lengthwise dark parallel-veined. A tri-partite yellow to white palate is only weakly developed (Fig. 1H, i.e. non clapper-like as, for example, in *P. calyptrata* – Fig. 1I) and placed immediately behind the corolla lower-lip middle lobe (that appears as a shallow yellowish-greenish shimmering dent on the ventral tube-side directly behind the base of the middle lobe).

To illustrate the differences that can be observed on herbarium specimens, we have chosen *Pinguicula calyptrata* (Bussmann et al. 16447 MO, barcode: MO 6589755, Fig. 2C) collected in the immediate neighbourhood of the *P. rosmarieae*-type population (Fig. 2A). The sheet shows three well-preserved, single-scaped flowering specimens with the flowers in lateral profile view. The leaf rosette with its curled-up leaf margins measures up to ~30 mm in diameter. The flowering scapes are up to ~50 mm tall; the flowers are up to ~12 mm long (spur included), the corolla is distinctly tri-partite into the lip, tube and spur; its lobes are nearly equal-sized (subisolobate), the lower-lip middle lobe is only slightly larger than the lateral lobes, ~5 mm long; the corolla tube is distinctly funnel-shaped (at throat widest, ~5 mm), about as long as the corolla lobes, ~5 mm long; the spur is distinctly separated from the tube, short, ~2.5 mm long, thin, at apex rounded (sometimes almost imperceptibly thickened), angled at about 60°–90° with the length axis of the tube. Overall, in *P. calyptrata* as in the other Andean *Pinguicula*-taxa, the corolla is distinctly divided into three parts: lip, tube and angled spur.

Contrarily, in *P. rosmarieae*, the corolla appears bi-partite: tube and spur form a straight uniform funnel-cone-shaped tube-spur-complex, i.e. the spur is not angled with the tube. These features are clearly visible on the specimen collected by Paníagua-Zambrana, Bussmann & Vega Ocaña 8586 (MO 6607881; Fig. 2B), gathered in the surroundings of the Laguna Huay(ll)abamba (Department San Martín). The plants were found growing either as lithophytes on a vertical rock wall in the spray of a small waterfall or as epiphytes on twigs of *Polylepis multijuga*. They are distinguished by a spread-out 6–8 leaved rosette appressed to the ground. Its flower shows the striking straight uniform tube-spur-complex which we also observed in the Laguna de los Cóndores-*Pinguicula* (see the three specimens on the left-hand side of the sheet). Photographs (Fig. 1H, I) of the corollas (frontal view) support the deep morphological disparity between the two taxa.

In *P. rosmarieae* (Fig. 1H), the corolla is widely open and appears radially symmetrical at first sight. The corolla lobes are spread out, the two lobes of the upper lip are smaller than those of the lower-lip, their distal margins are shallowly notched (to ~1/6 of their length). The lower-lip lobes are much larger, deeply notched (to ~¼ –1/3 of their length). The throat and the adjacent tube portion are not dark, a pronounced palate is not developed; it is replaced by a weak yellowish shimmering patch at the base of the corolla lower-lip middle lobe, continuing proximally (to the middle of the tube-spur-complex) lengthwise in two white hairy stripes.

In *P. calyptrata* (Fig. 1I), the corolla is widely open and also appears radially symmetrical. The corolla lobes are spread out, covering each other slightly with their lateral margins. They are nearly equal in size, except for the middle-lobe of the lower-lip that dominates the corolla to a certain degree. The distal margins of all corolla lobes are only shallowly notched, the throat being dark-purple coloured. Nearly 2 mm behind (proximally) the middle lobe, at the base of the lower-lip, a pronounced yellow clapper-like palate is inserted, from which two white hairy stripes stretch out into the tube.

### Habitat

The Departments Amazonas and San Martín in northern Peru partly occupy the Sierra zone between the dry coastal region (Costa) and the upper Amazon river lowlands (Selva) and are largely characterised by extensive and very species-rich, cloud forests and wet subalpine grasslands (páramos). In contrast to *Pinguicula calyptrata*, which is largely found on wet, often peaty, soils, in the páramo region, *Pinguicula rosmarieae* occupies a completely different, even wetter, habitat. The species has been found either growing on steep, often vertical, rock-walls, normally on sandstones, in the spray of waterfalls (Fig. 3A, B) or rarely as an epiphyte in dense moss layers on *Polylepis multijuga* (Fig. 3C). Both represent equally extreme habitats, with extremely wet, nutrient-poor and acidic conditions and considerable mechanical stress.

The population at the type location grew in full sunlight on a steep sandstone cliff immediately above the famous tombs built by the Chachapoyas culture (AD ca. 800–1500). The tombs were built underneath natural overhangs, thereby allowing dry storage of the mummies. The type population (Henning & Schneider 275) grows above these overhangs exposed to constant dripping water and the general high precipitation typical for the eastern slopes of the Andes in this region (Fig. 3A).

### Distribution

*Pinguicula rosmarieae* is endemic to the northernmost foothills of the Cordillera Central in northern Peru. The herbarium specimens, known so far, are from three nearby collections in the same mountain range southeast of Leymebamba, stretching over some 15 km in a north-south direction. Both expedition teams have mistakenly located the collection sites in the adjacent western Departments (Amazonas and La Libertad, respectively), since the border runs along the pass of the Cordillera. According to current online map-sources (Google maps), all populations, deposited in herbaria so far, were found some 4–6 km east of the border to San Martín (Fig. 4). The presumed narrow endemism has just recently been rebutted by the report of the new taxon from the Province of Bongará in the Department Amazonas some 100 km further north. Photographs made by L. Santa Cruz Cervera clearly show the characteristic rosette and flower patterns of *P. rosmarieae*, photographed in a similar open habitat near the famous Gocta falls. However, the taxon seems restricted to the northern branches of the Cordillera Central, but reaches further into the northernmost foothills. The collection data indicate that *P. calyptrata* and *P. rosmarieae* show distributional overlap over the entire range of the latter. True sympatry is nevertheless prevented by the different habitat requirements of the two taxa.

The area lies well in the so-called Amotape-Huancabamba Zone, an important biodiversity-hotspot that spans from the Pacific coast over the cordilleras to the tropical lowlands of southern Ecuador and large parts of northern Peru (Fig. 4, for details see: Weigend 2002, 2004;). Many plant groups have a centre of diversity here (Weigend et al. 2005; Struwe et al. 2009; Deanna et al. 2018) and show a concentrated occurrence of narrow-endemic taxa in that region (Berry 1982; Ayers 1999; Weigend 2002; Henning and Weigend 2009, 2009a; Henning et al. 2019). While the vegetation and flora of the inner-Andean valleys and the western slopes are relatively well-investigated, the eastern flanks of the Cordillera Central facing Amazonia are still under-collected in many areas. Especially, the areas east of Leymebamba (Dept. Amazonas) in the north and Buldibuyo (Dept. La Libertad) in the south remain largely unexplored, since the eastern slopes can only be reached by foot. Single collection trips have yielded unexpected, supposedly narrowly endemic taxa in other plant groups, although only a tiny fraction of the area could be sampled to date (e.g. Loasaceae: *Nasa rugosa* subspp. *gracilipes* and *pygmaea*; Henning et al. 2011).

The whole region is characterised by great geological diversity, with large areas of dolomitic karst, caused by the exceptionally high rainfall (the indigenous Chachapoya were often called “warriors of the clouds”), interspersed with small areas of sandstone outcrops and metamorphic rocks. The vegetation of the sandstone areas is particularly intriguing, with many endemic species and unique vegetation types (e.g. *Weinmannia* sp. and *Polylepis multijuga-Iochroma stenanthum* dominated forests). *P. rosmarieae* has been found exclusively in the spray of waterfalls on sandstone cliffs and as an epiphyte on *Polylepis* – both systems characterised by extreme moisture and almost permanent cloud cover, a fact that is recognised in the local topographic maps, which often simply indicate “clouds” in these areas.

*Pinguicula* is yet another example of a plant group that is present in the Amotape-Huancabamba Zone with an endemic taxon, whereas its widespread relatives have their distribution limits at the zonal boundary, either seen from within (*P. calyptrata*) or outside (*P. involuta*). Since *Pinguicula rosmarieae* has been collected in those areas of the overall region that are comparatively easy to access, it cannot be ruled out that there are additional taxa awaiting discovery. However, the nondescript shape of the insectivorous butterwort, represented by only a few, widespread taxa outside of that region (Fig. 4), does not encourage gathering.

On a broader scale, the endemism of *P. rosmarieae* is by no means a unique phenomenon amongst the South American *Pinguicula*. *P. jarmilae* Halda & Malina from southeast Bolivia (Department Chuquisaca, municipio Villa Serrano, village Nuevo Mundo), for example, is only known from the type locality (Halda et al. 2007). Although it grows on vertical sandstone (laterite) rock faces under flowing/moving water, its morphology is quite different from that of *P. rosmarieae*: the superficial similarity is likely coincidental and does not reflect a close relationship between the two taxa.

### Preliminary conservation status

Due to the aforementioned lack of a continuous botanical exploration of the region, we have to consider *P. rosmarieae* as Data Deficient (DD), according to the IUCN threatened species assessment guidelines (2001, 2017). However, within its distributional range, it seems to be limited to the easternmost ridge of the Cordillera Central. As an insectivorous plant, it seems to be adapted to special habitat types, characterised by ‘open’ vegetation, high precipitation and nitrogen-poor soils. These habitats might only be available in a narrow altitudinal band along the mountain chain. As our observations suggest, such sites are not present at similar altitudes in the west and it is very likely that suitable habitats are only available a few kilometres further east up to the lower altitudes of the Andes. This might indicate that *P. rosmarieae* is generally a rare taxon and might, thus, be vulnerable to habitat destruction. The lack of additional collections emphasises this assumption. This delicate butterwort might be in danger of extinction, but more data is needed to better assess the entire distribution and abundance, especially to the east of the known populations.

### Annotated list of *P. rosmarieae* specimens (paratypes)

**Peru**. “Dept. Amazonas: Distr. Chachapoyas” (sic!) = Dept. **San Martín**, Prov. Huallaga, Lei[y]mebamba, Oseres [10 km east of Leymebamba]. Bosque Montano, 2542 m a.s.l. (~06°58'S, ~77°40'W), 22 May 2015. *C. Vega Ocaña, L. Cotrina P., J. Valle, R. W. Bussmann, & N. Paniagua Zambrana 247* – HAO, MO 2852736. dp! [Det. *Pinguicula* RBU 2015; *involuta* E. Feltz (Ma), 2016]. – Duplicate: MO 6726506. “Dept. La Libertad, Distr. Uchumarca” (sic!) = Dept. San Martín, Prov. Huallaga, páramo and sandstone cliffs east of Laguna Huay(ll)abamba (06°58'53"S, 77°43'09"W) 3250 m a.s.l., 02 Nov 2012. – *N. Paníagua Zambrana, R. W. Bussmann & C. Vega Ocaña 8586* [det. *Pinguicula involuta* RBU 2015]. – MO 6607881. dp!

### List of *P. calyptrata* specimens in the research region (used for Fig. 4)

**Peru**: Dept. **Piura**: [Prov.] Huancabamba, Lomas Redonda (Sapalache-Chinguelas) 2400 m (05°09'S, 79°26'W) a.s.l., 15 Ago 1981. *A. Sagástegui Alva & al. 10187* – MO 2940293 [Barcode 348636], MO 4025844 [Barcode 348635] (dp!), HUT (dp!) – (det. *P. involuta* P. Taylor & M. Cheek). Dept. **Lambayeque**: [Prov.] Ferreñafe, [Distr.] Incahuasi cerca a la laguna Tembladera. Vegetación de jalca, zonas humedas. 3300 m a.s.l. (~06°09'S, ~79°19'W). 08 Oct 1989. *S. Llatas Quiroz 2606* – F 2051195 (dp!). (det. *P. involuta* P. Taylor 1992). Dept. **Cajamarca**: Prov. San Ignacio, Distr. Tabaconas, Local. Santuario Nac. Tabaconas-Namballe, alrededores de las lagunas Coyona (Arrebiatadas), 05°14'S, 79°16'W (Arriba Laguna Lagartocha), 3140 m–3180 m a.s.l.. *S. M. Baldéon Malpartida 5108 & L. Adriazon Ocupa*. – USM 00266675 dp! (det. *P. involuta*). – Cajamarca, km 30 de la carretera Cajamarca-Bambamarca Jalca, estepa de gramineas, sobre un afloramiento rocoso. 3600 m a.s.l., 23 Mar 1985. – *Sánchez-Vega, I. M., U. Molau & L. Ohmann 3756.* – F 2216084 (Barcode V0469923F - dupl. CPUN; dp! det. *Pinguicula* L.) – [Prov.] Santa Cruz, [Distr.] Pulán, [caserio] El Molino, 2,500 m a.s.l. (06°46'S, 78°55'W), 12 Feb 2007. *L. Santa Cruz, M. Chocce & M. Beltrán 996*– USM 241552 (dp!), HUT 50771 (dp!). – [Prov.] Santa Cruz, [Distr.] Pulán, Pampa el suro, 2500 m a.s.l. (06°50'S, ~78°54'W), 31 Ene 2008. *L. Santa Cruz 2098* – HAO, USM 240752 (dp!). – Prov. San Miguel, distrito Tongod, Bosque San Pedro Norte (06°45'S, 78°49'W), 03 Nov 2001. *I. M. Sánchez Vega & M. Sánchez M. 11122* – F 2245015 [V 0410057F] (dp! det. *P. involuta*). Dept. **Amazonas**: [Prov.] Bagua, [Santuario Nacional] Cordillera [de] Colan-La Peca, 9600 ft a.s.l. (05°35'S, 78°14'W), 29 Aug 1978. *Ph. J. Barbour 3222* – USM 53585 (pd!), MO 2798203 (dp! – Barcode MO 348631, det. *P. involuta* P. Taylor 1992). – Cordillera Colan-La Peca, 9600–11075 ft a.s.l. (05°35'S, 78°16'W), 08 Sep 1978. Ph. J. Barbour 3425 – F 1909251 (V0469936F; dp!), MO 2789874 (pd! det. *P. involuta* Taylor). – Amazonas: 3000–4000 m a.s.l. (~06°07'S, ~77°39'W), 09 Nov 2012. *H. van der Werff, L. Valenzuela, G. Shareva & A. Reyes Barrantes 25401* – MO. – Cerro de Fraijaca (Huaui-Huni) n. e. Tambo de Ventilla. Jul 07 1948. *A. W. Pennell 15875* USM 90946 (det. Pinguicula - dp!). – Prov. Chachapoyas, declives superiors de [Cerro] Puma-Urcú, [~2 km] este-sureste de [ciudad] Chachapoyas (~06°14'S, ~77°52'W), alt. 2700 m. – 3000 m., Jun 01 1962. *J. J. Wurdack 679*. – USM 90948 (dp!), COL (dupl. dp! det. *P. antarctica*, by Fernandes-Pérez 1964), NY. – Prov. Chachapoyas, bosque bajo y húmedo al lado de (moist scrub forest on south side of) Molinopampa-Diosan pass, alt. 2700 m – Balsa road to Leymebamba, just below Abra Callacalla (= Alba Barro Negro) on the slope towards Leymebamba, 3559 m a.s.l. (06°44'S, 77°53'W), 19.10.2000 – *M. Weigend, E. Rodríguez R., H. Förther & N. Dostert 867* – USM 166766 (dp! det. *Pinguicula* spec.) – [Prov.] Chachapoyas, [Distr.] Balsas, En el Paso de Calla Calla (06°48'S, 77°53'W), 07 Oct 2001. *I. M. Sãnchez-Vega, M. Sãnchez, M. 11061* – F. – Cerros [Cordillera] Calla Calla. 26 km above Leimebamba, road to Balsas. Km 403, 3360 m a.s.l. (~06°48'S, ~77°53'W), 16 Oct 1964. *P. C. Hutchison & J. K. Wright 6993*. – MO 2233627 (GBIF: as *P. antarctica*); USM 90947 (dp!). – Prov. Chachapoyas, Distr. Chachapoyas. Trail to Laguna de Los Cóndores, surroundings of Laguna Esperanza/Siete Lagunas (06°49'S, 77°43'W) 3275 m–3500 m a.s.l., Jun 26 2010. *R. W. Bussmann, A. Glenn, G. Chait & C. Vega Ocaña 16447*. Dept. **La Libertad**: Distr. Uchumarca, páramo in the surroundings of Vira Vira/Lagunas La Quinuas (07°00'S, 77°45'W), 3050 m a.s.l. – Photograph: *R. W. Bussmann* (det *P. involuta* = *P. calyptrata*).

## Supplementary Material

XML Treatment for Pinguicula rosmarieae
